# Supplementary material for: Oleaginous Heterotrophic Dinoflagellates—Crypthecodiniaceae
Source: Mar Drugs. 2023 Feb 28;21(3):162. doi: 10.3390/md21030162 (PMC10055936; doi:10.3390/md21030162)
Supplement: Supplementary file 1 [file marinedrugs-21-00162-s001.zip › Supplementary Tables S1 and S2.pdf]

Table S1. Primers.

Target region	Primer	Sequence (5′ to 3′)
LSU rDNA	LSU_D1R–F	ACCCGCTGAATTTAAGCATA
	LSU_R2–R	ATTCGGCAGGTGAGTTGTTAC
SSU rDNA	SSU_SR1–F	TACCTGGTTGATCCTGCCAG
	SSU_SR12b–R	CGGAAACCTTGTTACGACTTCTCC
ITS	ITS_SR12cF	TAGAGGAAGGAGAAGTCGTAA
	ITSrev	ATATGCTTAAATTCAGCGGGT
Cc18	Cc18-F	CAGTTCCTGAAGTTATTCGGCTCC
	Cc18-R	TGGGATGTCGCTGGGGTC
Cc20	Cc20-F	TGCAGACTGAATCCTTTGGC
	Cc20-R	GCTTTTGGAGAGACTACTACAGG

Table S2. Genbank accession numbers of the dinoflagellate rDNA sequences in phylogenetic tree analyses.

Organism / Strain	GenBank accession number		
	SSU	LSU	ITS
Crypthecodinium cohnii ATCC30021	OQ184972 *	OQ184965 *	OQ190440 *
Crypthecodinium cohnii ATCC30336	FJ821501.1	-	-
Crypthecodinium cohnii ATCC30556	OQ184973 *	OQ184966 *	OQ190441 *
Crypthecodinium cohnii CC1	OQ184971 *	OQ184964 *	OQ190439 *
Crypthecodinium cohnii CCMP316	KM068037.1	FJ939575.1	FJ823534.1
Crypthecodinium sp. CAAE-CL2	DQ322643.1	-	-
Crypthecodinium sp. D31	AB811790.1	AB811791.1	-
Crypthecodinium sp. SUN	KY263646.1	-	-
Crypthecodinium ryukyu ISG40-1	AB871546	AB871537	AB871528.1
Crypthecodinium ryukyu ISK-1	AB871544	AB871535	AB871526.1
Crypthecodinium ryukyu ISK-2	AB871545	AB871536	AB871527.1
Crypthecodinium okinawan OKI5-1	AB871551	AB871542	AB871533.1
Crypthecodinium okinawan SS2-2	AB871552	AB871543	AB871534.1
Crypthecodinium okinawan SZ7-1	AB871550	AB871541	AB871532.1
Crypthecodinium okinawan SZ13-1	AB871547	AB871538	AB871529.1
Crypthecodinium okinawan SZ13-2	AB871548	AB871539	AB871530.1
Crypthecodinium okinawan SZ13-3	AB871549	AB871540	AB871531.1
Crypthecodinium croucheri HKUST-1001	OQ184974 *	OQ184967 *	OQ190442 *
Crypthecodinium croucheri HKUST-1002	OQ184975 *	OQ184968 *	OQ190443 *
Crypthecodinium croucheri HKUST-1003	OQ184976 *	OQ184969 *	OQ190444 *
Crypthecodinium croucheri HKUST-1006	OQ184977 *	OQ184970 *	OQ190445 *
Undescribed Crypthecodinales R6	OQ297750 *	OQ297748 *	OQ190446 *
Alexandrium catenella	AJ535392.1	DQ785887.1	KF924271.1
Amphidinium carterae	AF274251.1	AY460585.1	EU927575.1
Cryptoperidiniopsoid sp. brodyi	AF080097.1	-	-
Cryptoperidiniopsoid sp. C	AY251292.1	-	-
Cryptoperidiniopsoid sp. Folly C5	AY590481.1	AY590481.1	AY590481.1
Cryptoperidiniopsoid sp. H/V14	AY245690.1	AY245690.1	AY245690.1
Cryptoperidiniopsoid sp. N	AY251286.1	-	-

Cryptoperidiniopsoid sp. NOAA Beach	AY590486.1	AY590486.1	AY590486.1
Cryptoperidiniopsoid sp. PLO21	AY245691.1	AY245691.1	AY245691.1
Gymnodinium impudicum	DQ785884.2	MH732685.1	DQ785884.2
Gyrodinium dominans	FN669510.1	FN669510.1	FN669510.1
Gyrodinium instriatum	DQ084522.1	JN020160.1	JN020162.1
Gyrodinium jinhaense	MH665396.1	MH665396.1	MH665396.1
Gyrodinium lebouriae (undescribed casual name)	-	EF681914.2	-
Gyrodinium moestrupii	HE611580.1	HE611580.1	HE611580.1
Heterocapsa triquetra	AF022198.1	AF260401.1	AF527816.1
Karenia brevis	AF274259.1	AY355457.1	FJ823562.1
Karlodinium veneficum	KU314867.1	MG737362.1	KU314867.1
Lingulodinium polyedrum	AF274269.1	EF613357.1	EU532481.
Peridinium willei	AF274280.1	AF260384.1	-
Prorocentrum micans	AY585526.2	AF260377.1	EU927523.1
Protoperidinium minutum	GQ227501.1	GQ227502.1	-
Scrippsiella trochoidea	AF274277.1	AF260393.1	MK930533.1

* New sequences isolated from this study.
