# Supplementary figures and images for: Oleaginous Heterotrophic Dinoflagellates—Crypthecodiniaceae
Source: Mar Drugs. 2023 Feb 28;21(3):162. doi: 10.3390/md21030162 (PMC10055936; doi:10.3390/md21030162)

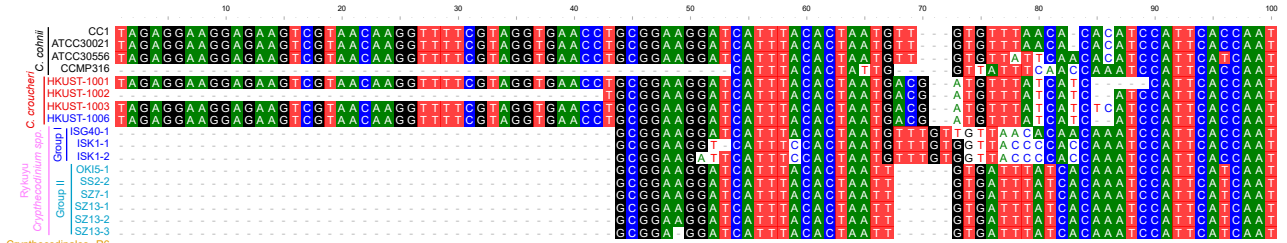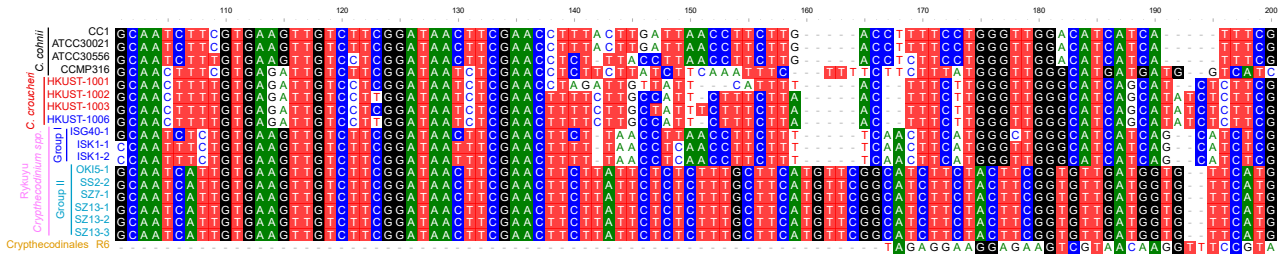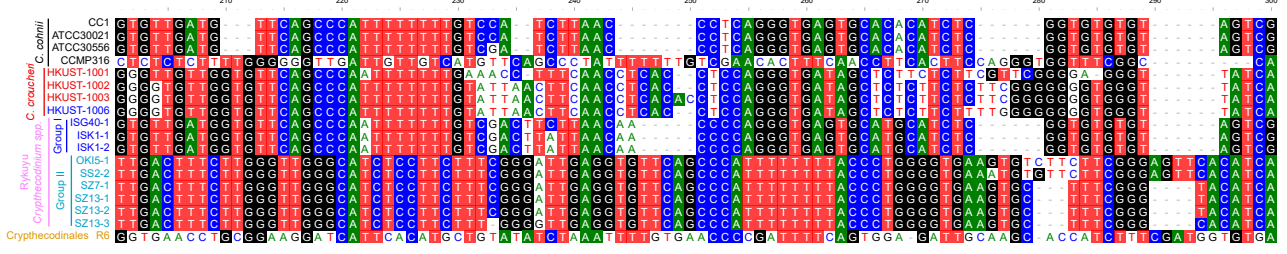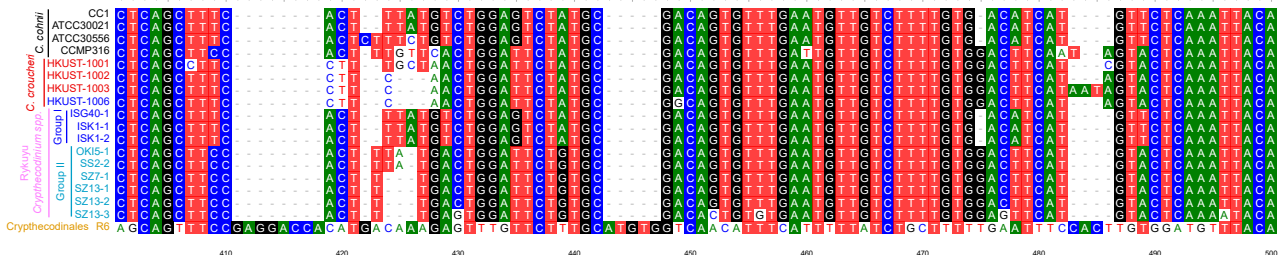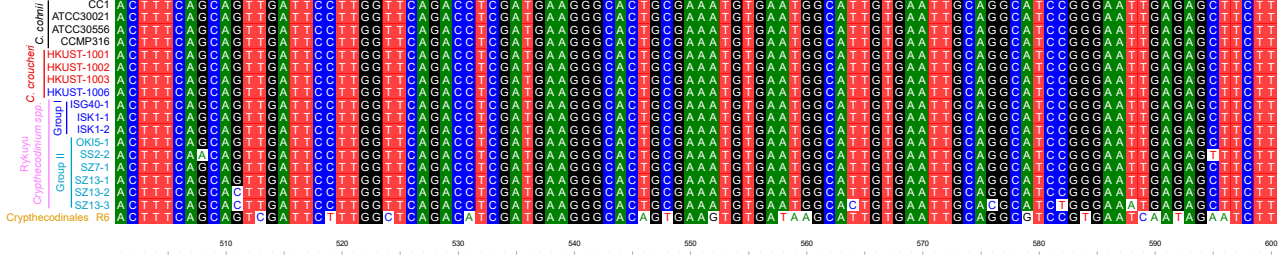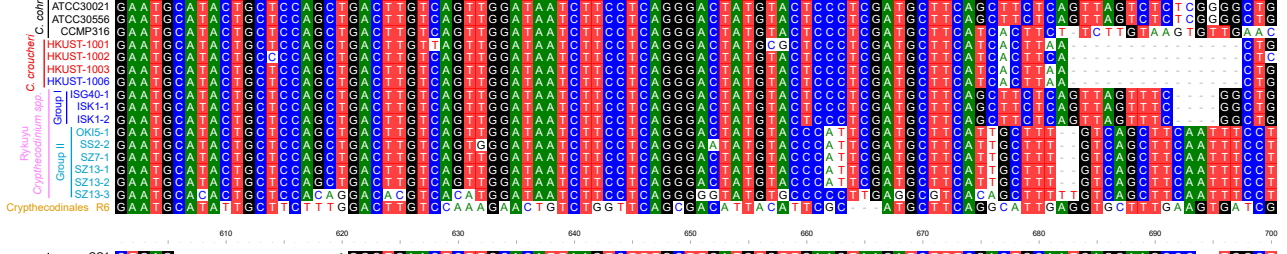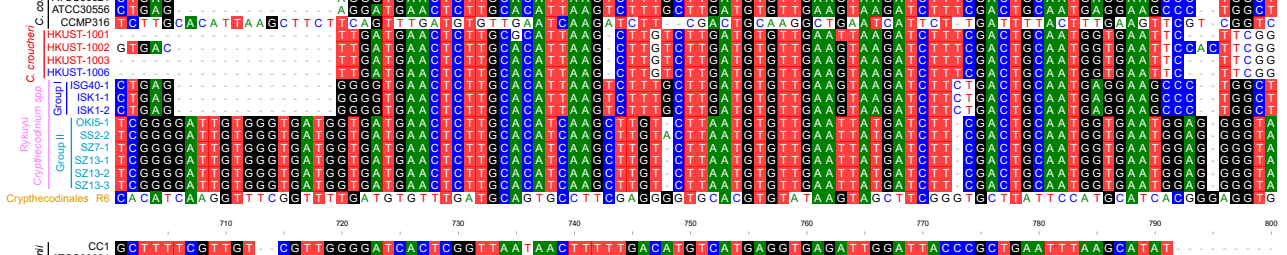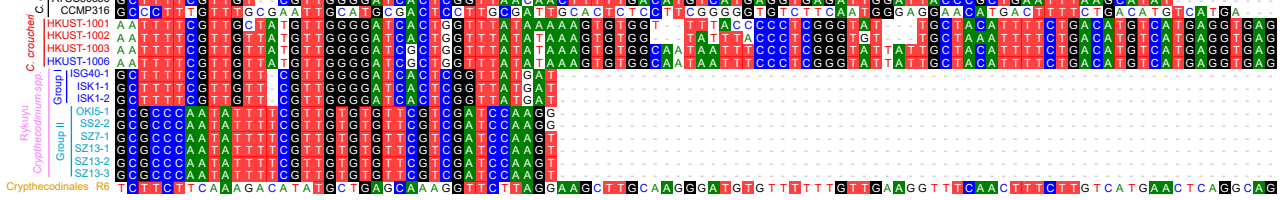

Supplement: Supplementary file 1 [file marinedrugs-21-00162-s001.zip › Figure S1.pdf]

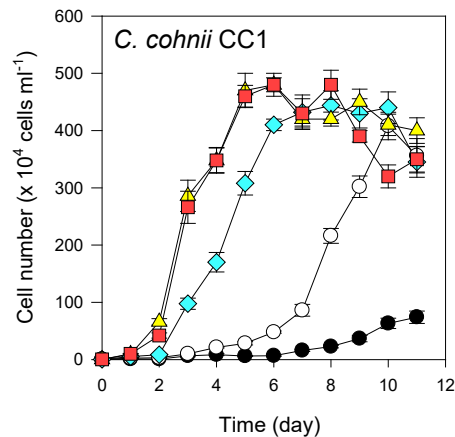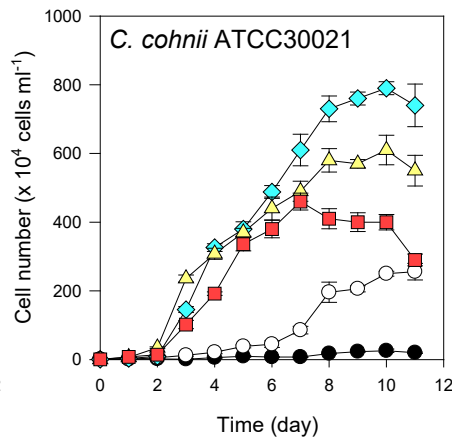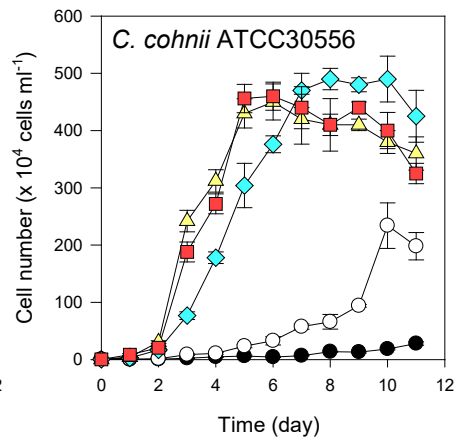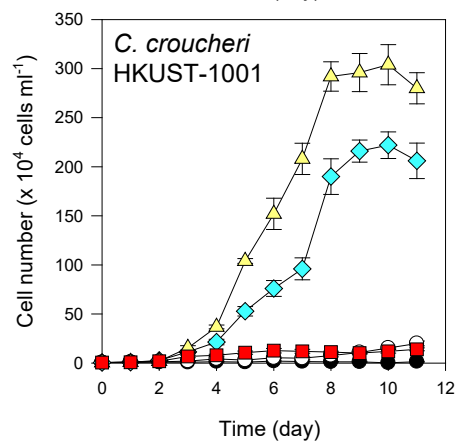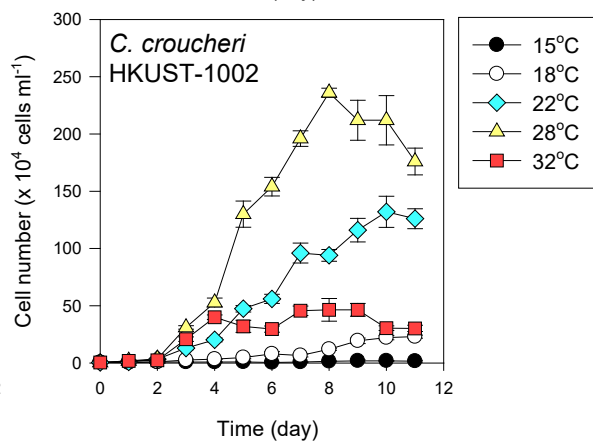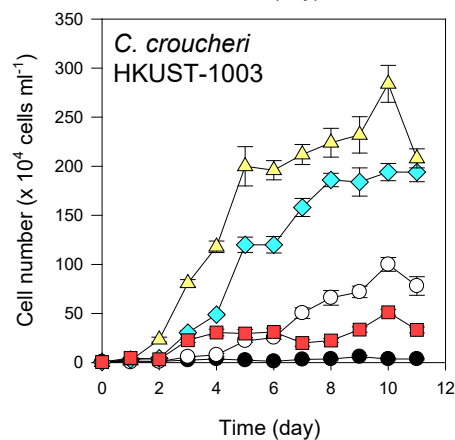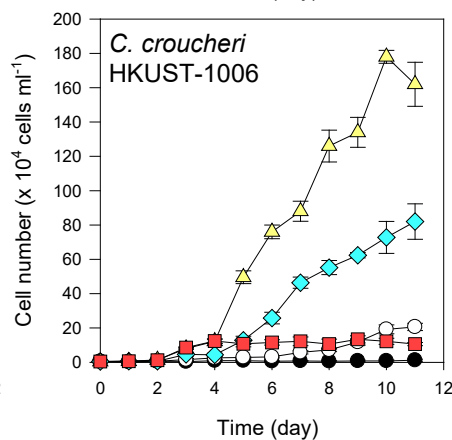

Supplement: Supplementary file 1 [file marinedrugs-21-00162-s001.zip › Figure S2.pdf]
